# A Premature Infant With Renal Tubular Dysgenesis Who Survived 61 Days With Anuria

**DOI:** 10.7759/cureus.86687

**Published:** 2025-06-24

**Authors:** Mari Hayata, Kei Takasawa, Eisuke Fukama, Ryo Aoki, Tomonori Ichikawa

**Affiliations:** 1 Pediatrics, Institute of Science Tokyo, Tokyo, JPN; 2 Pediatrics and Developmental Biology, Institute of Science Tokyo, Tokyo, JPN; 3 Neonatology, Japanese Red Cross Musashino Hospital, Tokyo, JPN; 4 Neonatology, Kawaguchi Municipal Medical Center, Saitama, JPN

**Keywords:** angiotensin-converting enzyme, anuria, autosomal recessive renal tubular dysgenesis, renal tubular dysgenesis, renin-angiotensin system

## Abstract

Autosomal recessive renal tubular dysgenesis (AR-RTD) is a rare and typically lethal disorder of the renin-angiotensin system (RAS), caused by mutations in genes such as angiotensinogen (*AGT*), renin (*REN*), angiotensin-converting enzyme (*ACE*), and angiotensin II receptor type 1 (*AGTR1*). It is characterized by severe hypotension, anuria, and features of Potter sequence, usually resulting in fetal or neonatal death. Although more than 15 surviving cases have been reported, survival has generally been associated with non-truncating mutations. We report the case of a premature male infant born at 34 weeks' gestation with clinical features of AR-RTD and confirmed homozygosity for a novel truncating *ACE* variant (c.2691delC, p.Phe898SerfsTer14). The pregnancy was complicated by oligohydramnios and fetal growth restriction, and the infant showed no urinary output throughout his life. Despite a poor prognosis, he survived for 61 days with intensive multidisciplinary support but without renal replacement therapy. Initial management included mechanical ventilation, nitric oxide, vasopressin for refractory hypotension, and strict restriction of fluid and electrolyte intake. Lactulose was administered enterally from day 32 of life to mitigate uremic symptoms, potentially aiding in the excretion of nitrogenous waste via the immature intestinal mucosa. The patient ultimately succumbed to renal failure on day 61 of life. Genetic testing confirmed the diagnosis, but parental testing was not performed. A literature review identified 61 genetically confirmed *ACE*-related AR-RTD cases, with an overall survival rate of 24.6%. Survival was significantly lower among patients with biallelic truncating mutations (8.6%) compared to those with at least one non-truncating allele. While truncating mutations were generally associated with poorer outcomes, exceptions existed, and variant location within the gene did not consistently predict prognosis. Interestingly, more reported mutations clustered on the 3’ end, although disease severity tended to be higher with 5’ variants. This case is notable as the longest known survival of a patient with confirmed AR-RTD and complete anuria, without renal replacement. The relatively prolonged survival may be attributed to the early initiation of supportive therapy immediately after birth, even before genetic confirmation. Importantly, the time gained allowed for meaningful genetic counseling and family bonding. This report underscores that survival beyond the neonatal period may be possible in AR-RTD cases with truncating *ACE* variants when early diagnosis and multidisciplinary management are initiated. It highlights the importance of prompt clinical suspicion and supportive care in maximizing life expectancy and enabling family-centered care, even in cases expected to have a dismal prognosis.

## Introduction

Autosomal recessive renal tubular dysgenesis (AR-RTD) is a rare genetic disorder caused by malfunction of the renin-angiotensin system (RAS). It is characterized by the absence or incomplete differentiation of the proximal tubule, which causes early-onset anuria, hypotension, and low reflux, leading to Potter sequence. It is associated with mutations in the four genes of the RAS, angiotensinogen (*AGT*), renin (*REN*), angiotensin-converting enzyme (*ACE*), and angiotensin II receptor type 1 (*AGTR1*) [1]. Mutations in these genes cause a lack of angiotensin II. Although the prognosis is poor, resulting in fetal or neonatal death [2], > 15 surviving cases have been reported (10 months to 19 years old, six with renal replacement therapy, five without renal replacement therapy). Although the presence of at least one non-truncating variant has been suggested to contribute to better survival and prognosis, surviving cases are not limited to those with non-truncating variants alone.

## Case presentation

A boy was born via cesarean section at 34 weeks of gestation because of oligohydramnios and fetal growth restriction. The patient’s medical history and family history of the parents were unremarkable. The parents were consanguineous (first cousins), and the pregnancy was spontaneous. The mother was 40 years old, gravida 5, para 4, and had one unaffected female child with her current husband. No medication or alcohol was administered during the gestational period, and no complications besides amniotic fluid deprivation or fetal growth retardation were noted during pregnancy.

**Figure 1:**
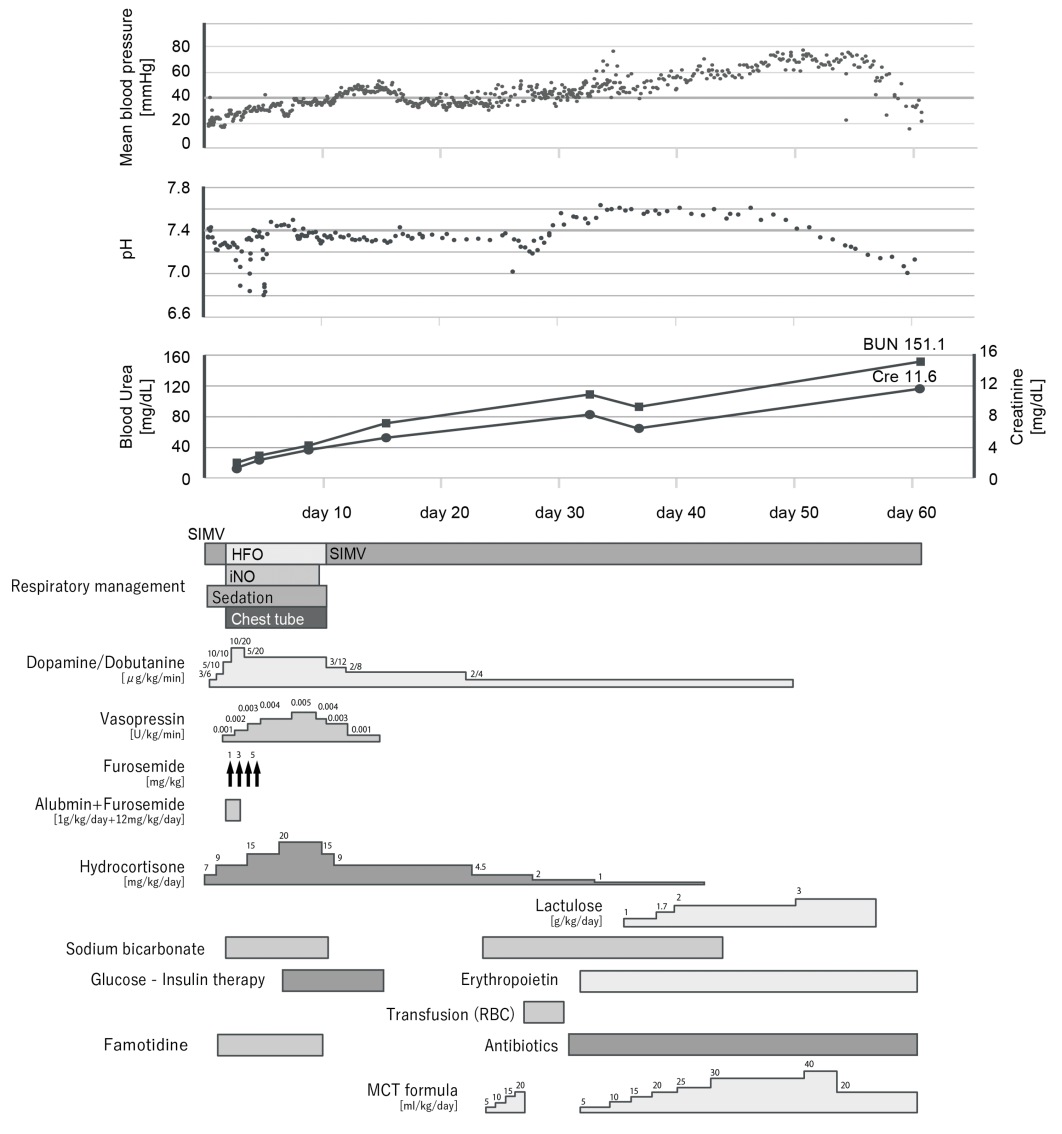
The clinical management of the patient. SIMV: synchronized intermittent mandatory ventilation, HFO: high-frequency oscillatory, iNO: nitric oxide inhalation therapy, RBC: red blood cell, MCT formula: medium chain triglycerides formula

The birth weight was 1,726 g (-1.8 SD), height was 41.0 cm (-1.5 SD), and head circumference was 29.3 cm (-1.2 SD). The Apgar scores were 3, 6, and 7 at 1, 5, and 10 minutes, respectively, and the cord artery blood pH was 7.336. The clinical course of the patient is shown in Figure 1.

**Figure 2:**
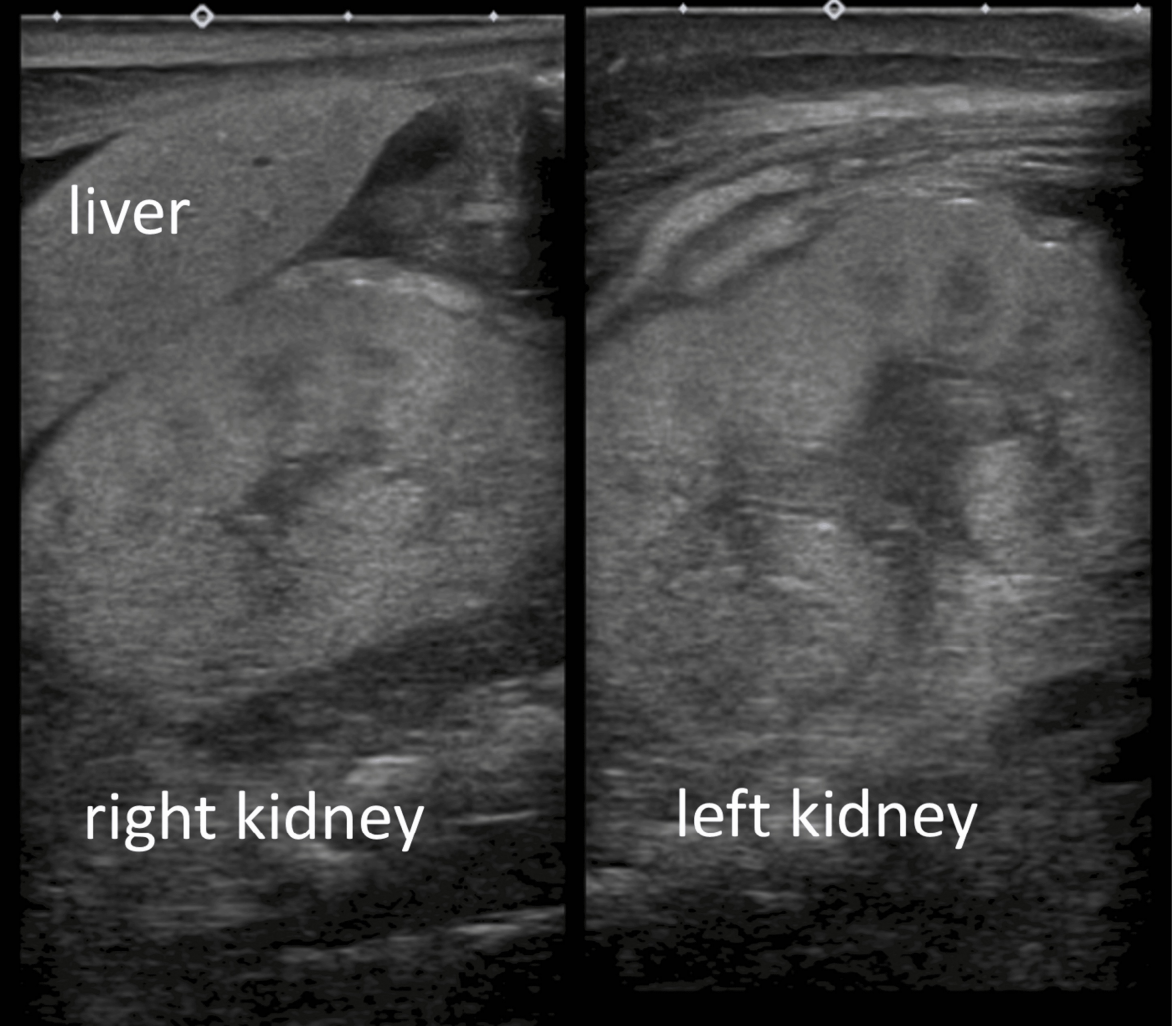
The ultrasound image of the kidney on admission. Both kidneys were highly echoic, with reduced corticomedullary differentiation.

**Figure 3:**
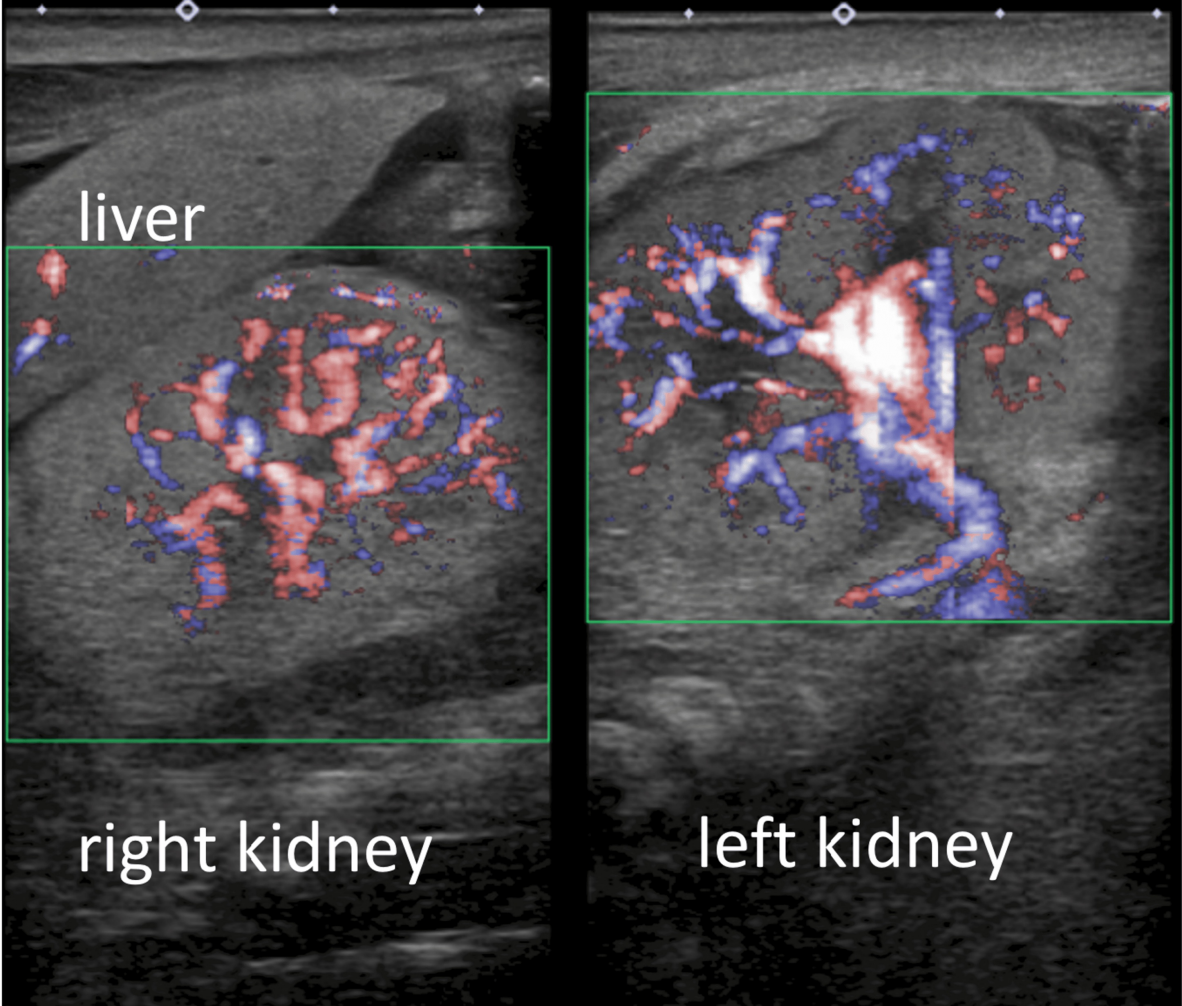
Color Doppler ultrasound on admission. The blood flow of both kidneys was normal.

The patient was intubated 10 minutes after birth because of respiratory distress. Wide cranial sutures, arthrogryposis, redundant skin, cryptorchidism, and micropenises have been observed. Radiography revealed pulmonary hypoplasia, and echocardiography revealed pulmonary hypertension without any congenital heart defects. The kidneys were normal in size, location, and shape on ultrasonography, with normal renal blood flow. However, they were highly echoic, with reduced corticomedullary differentiation (Figures 2-3).

These findings suggest the possibility of renal tubular dysgenesis; thus, fluid and electrolyte intakes were strictly restricted from day one. High-frequency oscillatory (HFO) ventilation, nitric oxide inhalation therapy, and sedation were initiated immediately after admission for pulmonary hypertension and respiratory distress. These interventions were discontinued on days of life (DOL) six, eight, and 10; however, mechanical ventilation was required throughout the patient’s life. Severe hypotension (mean arterial blood pressure < 20 mmHg) was resistant to high-dose epinephrine, dopamine, and hydrocortisone. The mean blood pressure elevated to 35-45 mmHg after vasopressin initiation on DOL two, and hypotension did not recur after vasopressin discontinuation on DOL 14. No urinary output was observed despite using high-dose diuresis (furosemide; 14 mg/kg/day) or hydrocortisone (20 mg/kg/day).

Multiple detailed counselling sessions were held, including detailed information regarding the prognosis and treatment options. The parents initially experienced a period of indecision, wavering between pursuing dialysis and choosing not to proceed. Ultimately, after thoughtful consideration, they decided against initiating dialysis. During this emotionally difficult time, there was a phase in which the parents, overwhelmed by grief and unable to fully accept the situation, temporarily stopped visiting the NICU. As their emotional state evolved, the medical team supported the family in spending time with their child in the ways they found most meaningful. This included holding the baby, engaging in skin-to-skin contact, and allowing visits with his three-year-old sibling. The care plan was continuously adjusted to respect and accommodate the parents’ wishes and needs, helping them build precious memories during their limited time together.

The creatinine and blood urea nitrogen levels increased from 1.29 mg/dL and 19.9 mg/dL on admission to 11.6 mg/dL and 151.1 mg/dL on DOL 60, respectively. Glucose-insulin therapy was initiated on DOL seven for hyperkalemia (K 7.5 mEq/L) and discontinued on DOL 15 without any recurrence of hyperkalemia. Enteral lactulose administration was initiated on DOL 32 to relieve uremic symptoms. Although lactulose is not a standard first-line treatment for uremia in neonates, the decision was made in consideration of the possibility that it might prolong the infant's survival, especially given that the family was still in the process of coming to terms with the child’s condition. Anemia with a low reticulocyte count and unresponsiveness to erythropoietin developed by the third week of life and required red blood cell transfusion. On DOL 61, the patient died owing to renal failure. The serum potassium remained at 6.2 mEq/L until death, making hyperkalemia less likely as the cause. Uremic encephalopathy was considered the most probable cause of death. Histopathological examination of the placenta revealed no fibrin deposition or abnormalities.

Based on the clinical findings, RTD was suspected and confirmed by genetic testing. A homozygote of a novel frameshift *ACE* variant (NM_000789.3:c.2691delC, NP_000780.1:p. Phe898SerfsTer14) was detected in exon 19. No other variants were detected in *ACE*, *AGTR1*, *AGT*, or *REN* by sequencing. Genetic testing of parents was not performed.

We obtained written informed consent for genetic analysis and publication this case report from the patient’s parents, in accordance with the Ethics Committee of the Institutional Review Board of Institute of Science Tokyo (Tokyo Medical and Dental University) (article#: G2000-103) and in adherence to the principles of the Declaration of Helsinki.

## Discussion

AR-RTD with confirmed *ACE* variants was extracted from the PubMed database. Patients who terminated the pregnancy were excluded. The results are presented in Table 1 and Figure 2. We identified 61 cases (15 surviving and 46 non-surviving cases) caused by 43 variants. The overall survival rate of the reported cases was 24.6%. The survival rates among the biallelic truncating variant, truncating and non-truncating cases, and biallelic non-truncating cases were 8.6%, 44.4%, and 47.1%, respectively.

**Table 1:** Survival rate among the reported cases. T: truncating variant, N: non-truncating variant

Variables	T/T	T/N	N/N	Total
Survival	3	4	8	15
Non-survival	32	5	9	46
Survival rate	8.6	44.4	47.1	24.6

Although AR-RTD remains fatal, > 15 surviving cases have been reported [3]. Two factors may determine the prognosis: (1) genetic background and (2) early intensive management and multidisciplinary treatment.

The survival rate of reported ACE-RTDs is lower in patients with truncating variants and has a genotype-phenotype correlation. Although several patients with biallelic truncating variants survived, the survival rate increased for at least one non-truncating variant. The variant type was related to severity, which is consistent with the results of previous reports.

**Figure 4:**
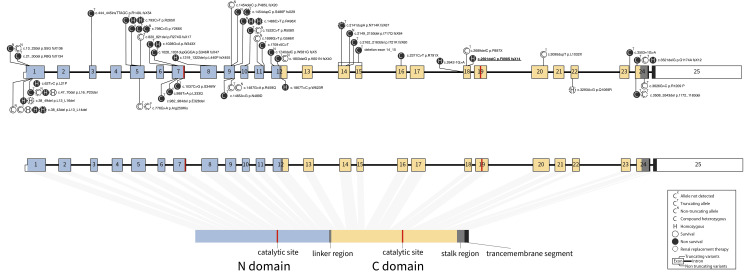
ACE structure and the reported variants. Truncating variants are shown over the gene, and non-truncating variants are shown under the gene. The present case is shown with an underline. ACE: angiotensin-converting enzyme

However, the prognosis cannot be predicted by the location or type of variation alone. Previously reported variants are shown in Figure 4 [1,3-18]. More variants are reported in 3’ side than in the 5’ side. Upstream variants are more likely to cause symptoms and develop diseases. However, some patients with non-truncating missense variants on the 5’ side took the severe courses.

The present case involved a homogeneous truncating variant and was expected to have the most severe prognosis. No urination was observed, which is consistent with the genetic diagnosis. However, the patient survived for two months. This is the longest survival case of an AR-RTD patient with anuria who did not undergo renal replacement. A possible reason for the two-month survival without any urinary output is that the variant was on the 5’ side. Moreover, the clinical diagnosis was made during the early postnatal period. This made it possible to initiate multidisciplinary treatment on DOL one without waiting for a genetic diagnosis, although the patient was not diagnosed during the fetal period. Water, nitrogen, and electrolyte intake were strictly restricted from DOL one. Previously, early postnatal vasopressin administration effectively stabilized blood pressure. Additionally, lactulose may affect the excretion of uremic toxins and water from premature intestinal mucosa with high permeability [19], as it promotes the fecal excretion of water, sodium, potassium, ammonia, urea, creatinine, and protons [20].

The two-month survival period allowed for genetic counselling based on early genetic diagnosis and provided time for the child to be with family members. This case shows that even children with anuria may survive beyond the neonatal period, depending on management, and that it may be possible to provide sufficient time for counselling and discussion of diagnosis, prognosis, and treatment with parents if similar children are born eventually.

## Conclusions

This case highlights the possibility of extended survival in infants with AR-RTD due to truncating* ACE* variants, even in the absence of urinary output and without renal replacement therapy. Early postnatal clinical diagnosis and prompt initiation of multidisciplinary management - including fluid restriction, vasopressin administration, and lactulose therapy - may contribute to prolonged survival, enabling time for genetic diagnosis, counseling, and family bonding. These findings suggest that individualized supportive care can offer meaningful benefits, even in cases previously considered uniformly fatal. We believe that these two months were essential for the family to process their emotions and ultimately reach a point where they could face the situation and spend meaningful time with their child.
